# RACIAL DIFFERENCES IN THE DAILY EXPERIENCES OF AFRICAN AMERICANS AND EUROPEAN AMERICANS PROVIDING CARE

**DOI:** 10.1093/geroni/igz038.2934

**Published:** 2019-11-08

**Authors:** Kelly E Cichy, Athena Koumoutzis

**Affiliations:** Kent State University, Kent, Ohio, United States

## Abstract

African Americans often report lower caregiver burden, however, few studies consider the broader daily context of African American caregivers’ lives. This study examines racial differences in the associations between providing care for a spouse or parent and daily health and well-being among African Americans and European Americans, including how other daily stressors moderate these associations. During eight days of interviews, respondents aged 34 to 84 years (N = 1,931) from the National Study of Daily Experiences (NSDE II) reported on their daily stressors, negative affect (NA), physical symptoms, and whether or not they provided support to a spouse or parent with a disability. Controlling for demographics, on caregiving days, NA was higher than on non-caregiving days (p < .05) for all respondents. On caregiving days with no work stressors, African Americans only reported more physical symptoms than on caregiving days with work stressors (p < .05). Implications will be discussed.

